# Nanocrystalline Iron Monosulfides Near Stoichiometry

**DOI:** 10.1038/s41598-018-24739-8

**Published:** 2018-04-26

**Authors:** Dennice M. Roberts, Alyssa R. Landin, Timothy G. Ritter, Joel D. Eaves, Conrad R. Stoldt

**Affiliations:** 1 Department of Mechanical Engineering, University of Colorado Boulder, Boulder, Colorado 80309 United States; 2 Department of Chemistry and Biochemistry, University of Colorado Boulder, Boulder, Colorado 80309 United States

## Abstract

Solids composed of iron and sulfur are earth abundant and nontoxic, and can exhibit interesting and technologically important optical, electronic, and magnetic phenomena. However, the iron-sulfur (Fe-S) phase diagram is congested in regions of slight non-stoichiometric iron vacancies, and even when the iron atomic composition changes by even a few percent at standard temperature and pressure, there are myriad stable crystal phases that form with qualitatively different electronic properties. Here, we synthesized and characterized nanocrystals of the pyrrhotite-4M structure (Fe_7_S_8_) in an anhydrous oleylamine solvent. Upon heating from 140 °C to 180 °C, the solid sequentially transformed into two kinetically trapped FeS intermediate phases before reaching the pyrrhotite-4M final product. Finally, we assessed the effects of iron vacancies using the stoichiometric end-member, troilite, as a reference system. Density functional theory calculations show that iron vacancies in troilite shift the structure from hexagonal FeS to a monoclinic structure, similar to crystal structures of pyrrhotites, and suggest that this iron deficient troilite may be a stable intermediate between the two crystal structures. The calculations predict that defects also close the band gap in iron deficient troilite.

## Introduction

Iron sulfide solids play central roles in biological processes, catalysis, planetary science, prebiotic chemistry and geochemistry^1–5^. Comprised of earth abundant and nontoxic elements, the optoelectronic and strongly correlated physics that these materials exhibit have a myriad of fundamental properties and technological applications. For stoichiometric FeS, the two crystal phases that have received the most attention are troilite and mackinawite. Troilite is predicted to exhibit large room temperature magnetoelectricity, while mackinawite is reported to be an unconventional iron-based superconductor, analogous to FeSe^6–9^. Understanding the boundaries between and properties of compositions in this phase space is critical not only for accessing such applications but for advancing the means by which we can explore and assess complicated, defect-intolerant materials. However, facile nature of the oxidation states and bonding patterns in both sulfur and iron makes these compounds difficult to synthesize, characterize, and model computationally relative to other strongly correlated iron-based materials^5,10–13^.

In many ways, the similarities that iron sulfide solids share with other complex and kinetically frustrated systems having several stable configurations, such as glasses, proteins, and colloids, are striking^14^. Where the phase diagram of these materials has been measured, iron sulfides exhibit a large diversity of polymorphs and polytypes that are separated by tens of Kelvin and single percentages of mole fraction^15–18^. Whether or not these compounds form structures that are thermodynamically separable and stable or that are instead kinetically isolated from one another remains an open question. Slight changes to the Fe-S composition ratios of these materials lead to large variations in structural, chemical, optoelectronic, and other physical properties^6,10,16,19,20^. Iron defects in these materials, even at a concentration of a few percent, affect their properties in poorly understood ways^21–23^. These complexities have plagued a comprehensive structural and chemical characterization of nearly stoichiometric FeS, as well as systems for which FeS is a precursor, namely iron pyrite (FeS_2_), a theoretically promising material for photovoltaic applications. Understanding and quantifying their nature systematically is a principal component of the work presented here, and is a necessary step in rational synthetic control over iron sulfide compounds in general.

At ambient pressure and temperature and in an anhydrous environment, stoichiometric FeS takes the form of troilite, a massively distorted, octahedrally coordinated NiAs-based crystal in the hexagonal *P-62c* space group with unique electronic properties arising from strong electron correlation. Ricci and Bousquet predict that troilite should have a large room-temperature magnetoelectric effect, while Guénon *et al*. have explored troilite as a candidate for a non-tetragonal high T_c_ iron based superconductor^8,24^. *In situ*, troilite samples are exclusively found in meteorites as a minority phase, and are thus seldom without impurities and defects. As a result, experimental work with natural samples cannot guarantee that results are free of compositional artifacts. Additionally, both synthetic and natural samples of the composition of FeS, measured at sufficiently high resolution, report that troilite contains iron vacancies^25–27^.

Pyrrhotites are a series of Fe-S structures possessing a NiAs-based structure, like troilite, but with slightly iron deficient compositions (Fe_1−x_S) that contain a host of variations in stoichiometry and unit cell geometry^28^. Reported instances of natural and synthetic Fe_1−x_S systems see iron deficiencies anywhere from 0.004 ≤ x ≤ 0.143^16,29^. This region of the Fe-S phase diagram is ill defined, not only in the scope of the structures it encompasses, but also in the transformational pathways between the different crystal structures. Reported compositions of iron deficient troilite have off-stoichiometries comparable to some pyrrhotites even though troilite technically denotes a stoichiometric FeS structure^26^.

The naming convention for pyrrhotites refers to its various superstructures and is generally presented as a number correlating to repeated layers and a letter which represents the axis along which the repetition occurs; for example, “5 C” or “4 C”. This family of materials primarily takes on monoclinic or hexagonal structures, and on occasion are distinguished further using “M” or “H” instead of “C” to denote monoclinic or hexagonal, respectively^15^. We note that less iron deficient structures tend to share the hexagonal crystal structure of troilite^5^. Magnetic and electronic properties also differ with these slight structural variations, particularly in relation to vacancy distributions that are driven by composition^19^.

In this work, we present a facile, non-aqueous synthetic route to nanocrystals of near stoichiometric pyrrhotite-4M structure, as well as the metastable intermediate compounds that form prior. We provide a comprehensive analysis of the crystal structure and morphology using x-ray diffraction (XRD), field emission scanning electron microscopy (FE-SEM), and high-resolution transmission electron microscopy (HRTEM). By employing electronic structure calculations using electronic density functional theory (DFT), we investigate, for the first time, the role of iron vacancy defects on structural and electronic properties of the near stoichiometric solid.

## Methods

### Experimental methods

Nanoscale pyrrhotite was synthesized using a 1:1 molar ratio of FeCl_2_ and elemental S precursors. All chemicals were from Sigma Aldrich and used without further refinement. In a three-necked flask, 0.5 mmol (63.372 mg) anhydrous FeCl_2_ was added to 20 mL oleylamine (OLA). The system was filled with argon and vacuum pumped for three cycles. After argon was returned to the system, 0.5 mmol (16.03 mg) elemental sulfur and 10 mL OLA was added; the system was held under vacuum again and stirred for 2 minutes at a medium rate with a stir bar. Under argon and with continued stirring, the system was brought up to 180 °C for 2 hours (unless otherwise noted) and then cooled naturally to room temperature. Nanoparticles were washed three times via centrifugation at 3000 RPM with a mixture of methanol and chloroform. Material was stored either in chloroform or as a powder in a glovebox to maintain integrity and minimize air contact.

X-ray diffraction data was taken using a D2 Phaser diffractometer using a Cu K-alpha radiation source with wavelength 0.154056 nm. Samples were prepared by two different methods: (1) nanoparticles in solution were pipetted onto a silicon zero diffraction plate and the solvent was allowed to evaporate, and (2) nanoparticles stored in a glovebox as a dry powder were loaded in a home-built air-free sample holder covered in Be foil.

High resolution TEM was collected at the University of Colorado Boulder via the Molecular, Cellular, and Developmental Biology department. A FEI Tecnai F-20 at 200 kV was utilized; images were captured using a Gatan Ultrascan US-4000 4 k × 4 k camera.

Scanning electron microscopy images were taken using a Hitachi SU 3500 microscope with a 10 kV working voltage, 4.7 mm working distance, and 40 k magnification. Particles were dropcast on a silicon plate and images were collected without coating or further processing.

Rietveld refinement was performed using the EXPGUI package of GSAS^30,31^. The crystallographic model for fitting was taken from the American Mineralogist Crystallography Database. Thermal and occupancy parameters remained fixed as laboratory XRD data is insufficient for parameters of this sensitivity. Background and instrument zero were refined and then fixed for the remainder of the process; fractional coordinates, cell, and profile parameters were sequentially refined. The fit quality parameters are as follows: X^2^ = 3.126, wR_p_ = 0.1548, R_p_ = 0.1200.

### Theoretical Methods

For the Fe-S systems studied here, there are two major computational challenges. First, the unit cells, particularly for defective structures, contain many electrons and adopt a priori unknown crystal structures that have low symmetry space groups. Second, FeS compounds exhibit strong electron correlation due to the presence of iron. These challenges require a computational method that can reasonably capture the effects of strong electron correlation, and is also computationally feasible for large periodic systems.

Because troilite is the end-group compound in this study, we use it as a computational reference system. We performed all DFT calculations using the Vienna *Ab initio* Simulation Package (VASP)^32–35^ and employed the exchange-correlation functional based on the Perdew Burke-Ernzerhof (PBE) form of the generalized gradient approximation (GGA)^36^, augmented with a rotationally-invariant Hubbard-like U term to account for strong “on-site” electron correlation on the iron 3d orbitals. Sometimes called the “DFT + U” method^37,38^, this methodology provides results for structural quantities, like lattice constants, that agree well with experimental measurements for the related iron sulfur compounds of troilite and iron pyrite^8,12,39^. The presence of an insulating state in our DFT + U calculations for troilite is consistent with calculations from the much more computationally expensive dynamical mean field theory (DMFT) methods, that also find an insulating ground state, but one with a much narrower gap^40,41^. Because the ground states of troilite and pyrrhotite are magnetic, all of the calculations reported here are spin-polarized. Our DFT + U calculations robustly reproduced the unusual magnetic structure of the ground electronic state in troilite, also consistent with the above DMFT calculations. The chosen DFT + U methodology is thus a compromise between computational tractability and accuracy that we expect to be able to find and compute accurate crystal structures and distinguish between gapped and ungapped electronic structures for several iron sulfur compounds.

**Figure 1:**
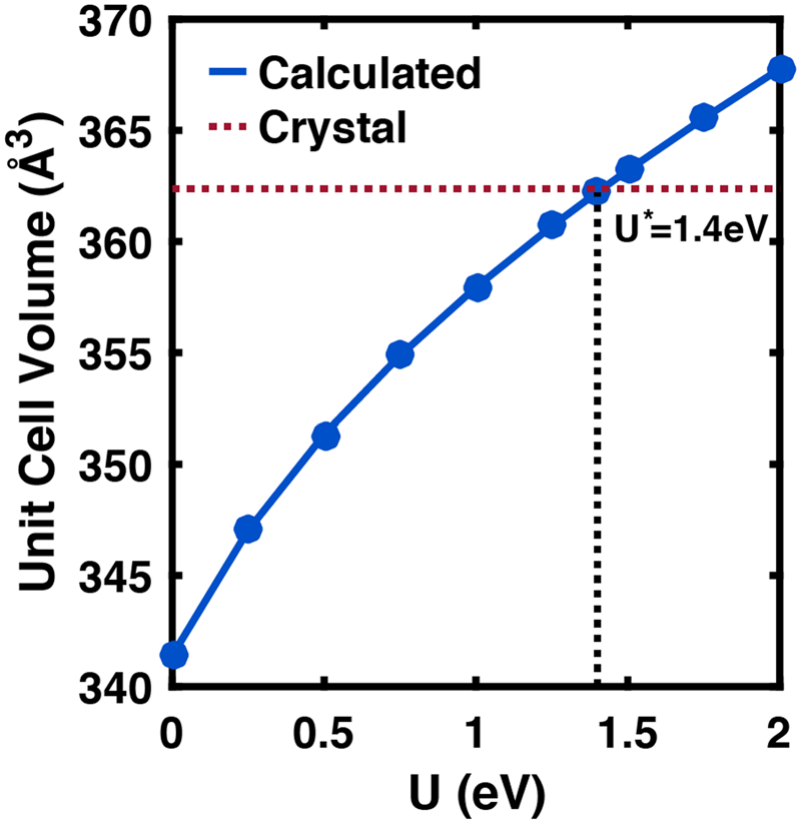
Relationship of U term used for DFT calculations and cell volume. U* represents the U value that most closely matches the experimental unit cell volume. We use this value for U in subsequent calculations.

In this DFT + U scheme, we determined the optimal value of U, defined to be U*, by comparison to the experimentally measured bulk unit cell volume in troilite^42^. The parameter we call U here is really U-J, with J = 0^38^. Figure 1 shows the monotonic increase in the unit cell volume of the fully relaxed structure as the value of U increases. Compared to previous calculations, our U* is slightly larger than some reports, but comparable to others^8,12,23^. The value U = 1.4 eV gives a hexagonal unit cell for troilite with lattice parameters *a* = *b* = 5.960 Å, *c* = 11.773 Å, a unit cell volume of 362.22 Å^3^, and matches experimental values for these parameters to within 0.5%. We take this value for U* to calculate the electronic properties in the Fe-deficient regime.

DFT results reported for troilite’s electronic properties are inconsistent, which suggest that the properties of FeS materials are sensitive to choices in DFT methodology, especially the choice of pseudopotentials. Some reports using the GGA class of functionals, but different pseudopotentials, calculate a slightly insulating state^8,20^, while others see no band gap^12,23,43,44^. Our choice of pseudopotentials, based on the projection augmented wave function (PAW) pseudopotentials^45,46^, are harder than those used in previous studies and incorporate more valence electrons. These potentials are therefore minimally approximate with respect to both the earlier studies and the all-atom orbitals. We used 6 valence electrons per sulfur atom, and 16 electrons for each iron. Hard pseudopotentials require a larger basis set than soft pseudopotentials, and thus come with an increase in computational cost. The plane wave energy cutoff was set to 700 eV, which ensured energy convergence to within 10 meV per unit cell.

We relaxed all troilite structures, defective and nondefective, starting from the nondefective experimental troilite crystal structure with 24 atoms in the unit cell. From this structure, we generated defective structures by removing one iron atom, which corresponds to a defect concentration of 1/12, or about 8.3 atomic percentage iron deficient. We checked that the reported structural and electronic results are insensitive to which atom gets removed by removing all 12 different iron atoms in the unit cell and repeating the structural minimization and density of states calculations. Using the conjugate gradient method, we moved the atomic positions and unit cell dimensions at constant pressure until the forces in each self-consistent cycle were less than 1 meV/Å. The wave vector spacing was based on a 6 × 6 × 3 Γ-centered Monkhorst-pack grid. For the electronic density of states (DOS) calculations in troilite, we used a 19 × 19 × 10 grid. Both grid choices sample the Wigner-Seitz cell evenly in all directions. For the pyrrhotite-4M DOS calculations, we used an 8 × 15 × 8 k-point grid and left the atomic positions at the experimentally determined values^11^. All energies were converged to within 1 × 10^−8^ eV in each self-consistency cycle using Gaussian smearing with a width of 0.01 eV and crystal structures in this article were generated and visualized using the XCrySDen visualization package^47^. The troilite and pyrrhotite unit cells were initialized in their magnetic ground states with ±4 μ_B_ and ±3 μ_B_ on each Fe ion, respectively, ferromagnetic alignment in the a-b plane, and antiferromagnetic alignment along the c axis^8,10,11,13,43^.

### Data Availability

The datasets generated during and/or analysed during the current study are available from the corresponding author on reasonable request.

## Results and Discussion

### Structural Transformations during Growth

The solvothermal synthesis applied here is adapted from a method previously used in the production of nanoscale solids^18,48^, and utilizes OLA for solvation of the precursor ions as well as to confine the growing FeS crystallites. Amine solvents such as OLA are soft bases, and as such, do not exhibit a strong affinity to metal ions such as Fe^2+ ^^49^. Therefore, this weak interaction is not expected to strongly moderate particle nucleation and growth rates. Previous geologic research work has shown the Fe-S system to evolve from nucleation through two intermediate phases before reaching the FeS or FeS_2_ phases in a hydrothermal environment^29,50,51^. First to form is the two-dimensional, metastable mackinawite (FeS) phase, followed with increasing temperature by the intermediate greigite (Fe_3_S_4_) phase. The kinetics of transformation are shown to follow zero-order kinetics, and are commensurate with a solid-state mechanism for interconversion^51^. Of particular note here is the role that the oxidation and reduction of iron plays during the transformation process, where the intermediate greigite phase is a mixed iron (II/III) spinel that derives from and then transforms into ideally Fe^2+^ containing compounds. We also note the unique nature of an anhydrous amine-based synthesis as opposed to hydrothermal syntheses, where water based approaches are generally considered difficult to control and reproduce^5^.

**Figure 2:**
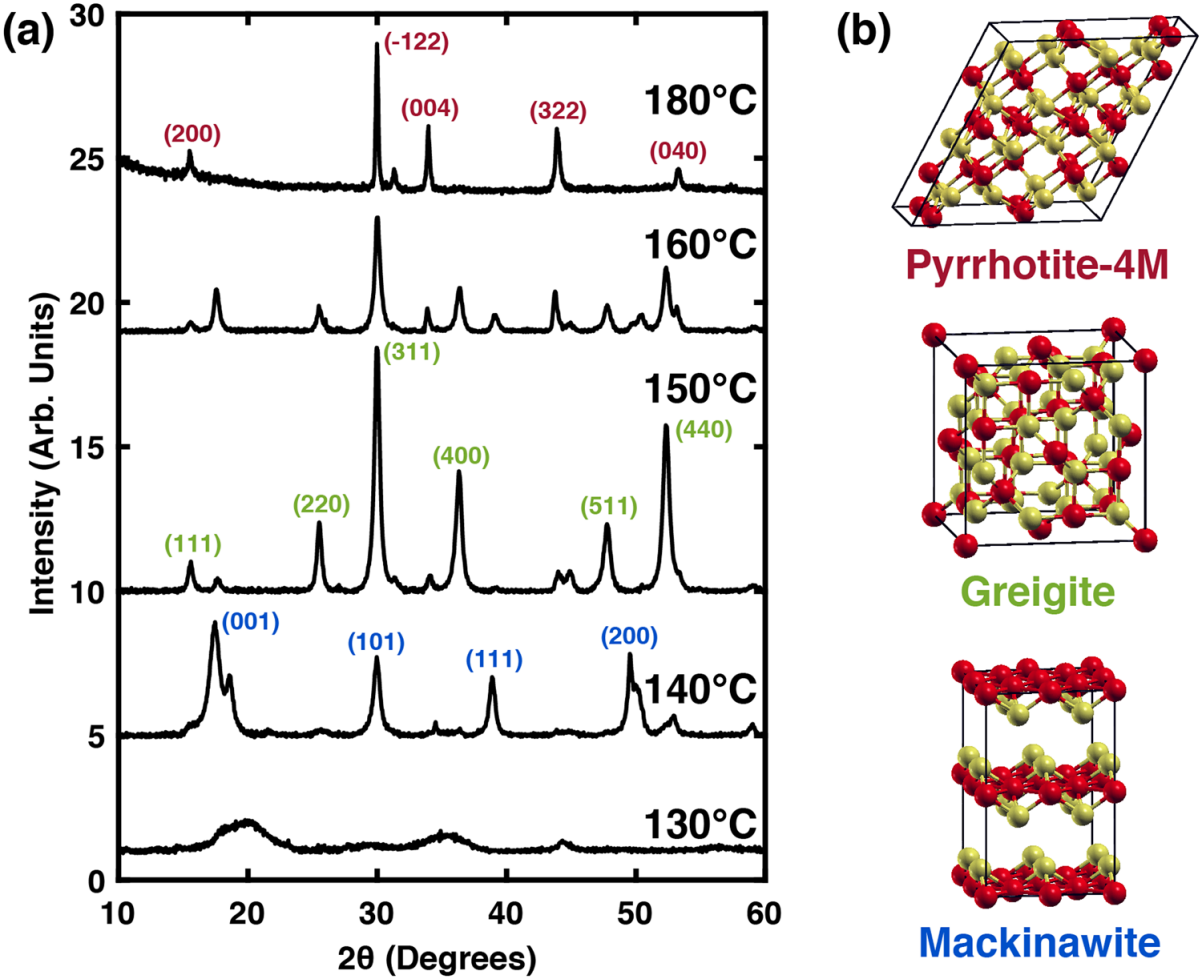
Room temperature X-ray diffraction patterns of FeS products synthesized at different temperatures. The spectra in (**a**) show diffraction patterns for products synthesized from 130 °C to 180 °C. Peak assignment colors correspond to the structures illustrated in (**b**) with iron and sulfur labeled red and yellow, respectively.

To explore the commonalities between the FeS and FeS_2_ stoichiometries, we first performed a study to determine if the monosulfide evolves along a similar trajectory in our reaction chemistry with increasing growth temperature as is observed for FeS_2_, and is similarly reported in hydrothermal synthetic environments^29^. To investigate this, reaction mixtures of 1:1 ratio of Fe to S were heated in OLA solvent under argon at temperatures from 120 to 180 °C for 120 minutes, and the respective products were characterized for crystal structure by powder XRD. Shown in Fig. 2, XRD patterns are compared for each growth temperature. At temperatures below 140 °C, the product of the synthesis is found to be amorphous, with no defined diffraction peaks present. At about 140 °C, the first evidence of crystalline mackinawite is observed in the diffraction profile. Mackinawite crystallizes in the tetragonal space group, *P4/nmm*, with the (001), (101), (111), and (200) diffraction peaks resolved in Fig. 2. Additional, less intense diffraction peaks are also observed at this temperature, and are indicative of the greigite and pyrrhotite phases beginning to form. Upon heating the 1:1 Fe to S mixture to 150 °C for 120 min., we observe new diffraction peaks associated with the transformation of mackinawite primarily into the cubic greigite phase with *Fd3m* symmetry. Here, we detect the (111), (220), (311), (400), (511), and (440) diffraction peaks associated with this phase, as labeled in Fig. 2.

Upon heating the 1:1 Fe to S mixture for 120 min. at 180 °C, we observe a nearly complete transformation of the intermediate FeS and Fe_3_S_4_ phases to the pyrrhotite-4M phase. Variation in peak intensities and peak broadening from the 4M reference pattern suggests additional minority phases may be present after 120 min., as other pyrrhotite polytypes such as Fe_9_S_11_ and Fe_13_S_16_, as well as stoichiometric troilite, have diffraction patterns that closely resemble Fe_7_S_8_ and thus are difficult to rule out completely. However, as stated previously, the complexity of the Fe-S system with low iron deficiency presents a diffraction pattern that is not readily deconvoluted. Therefore, in the next section, we describe the results of extending the heating time at 180 °C to 400 min. in order to more fully react the Fe-S system. At the intermediate temperatures between 140 and 180 °C, the XRD data shows coexistence of the three phases, with relative phase proportions being impacted by the chosen reaction conditions.

### Pyrrhotite Morphology and Structure Characterization

**Figure 3:**
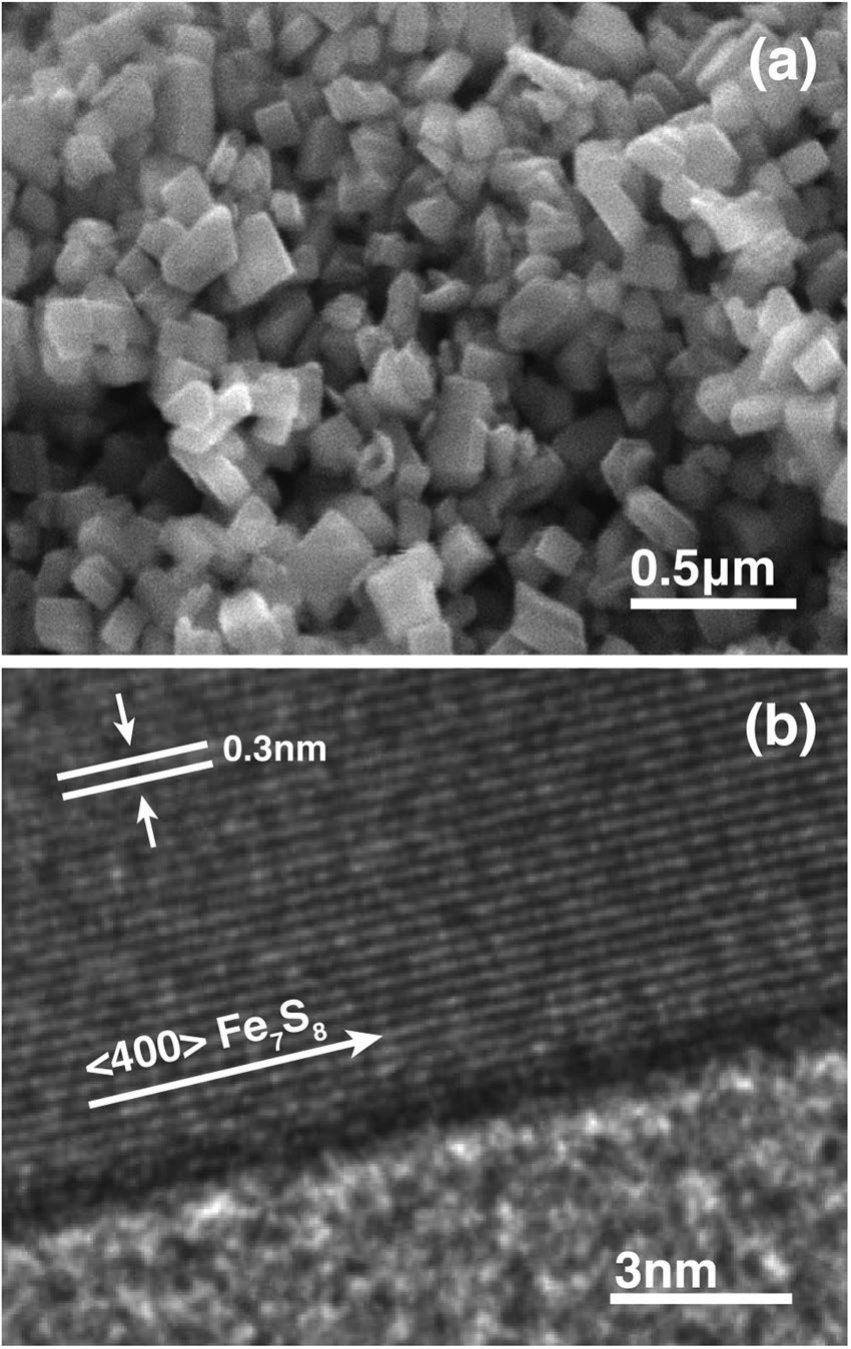
Images of as-synthesized nanoparticles illustrating morphology and unit cell spacing. Panel (a) shows FE-SEM crystallites and (**b**) uses HR-TEM to show individual lattice planes of sulfur atoms.

In Fig. 3a, a field emission scanning electron microscopy (FE-SEM) image of a dropcast pyrrhotite-4M product shows crystallites with sizes below 100 nm and a plate-like morphology that reflects its monoclinic symmetry. In Fig. 3b, a high-resolution transmission electron microscopy (HR-TEM) image is shown for the edge portion of a single crystallite. The measured lattice spacing of approximately 0.3 nm corresponds well with the parallel planes of sulfur atoms oriented along the <400> direction.

**Figure 4:**
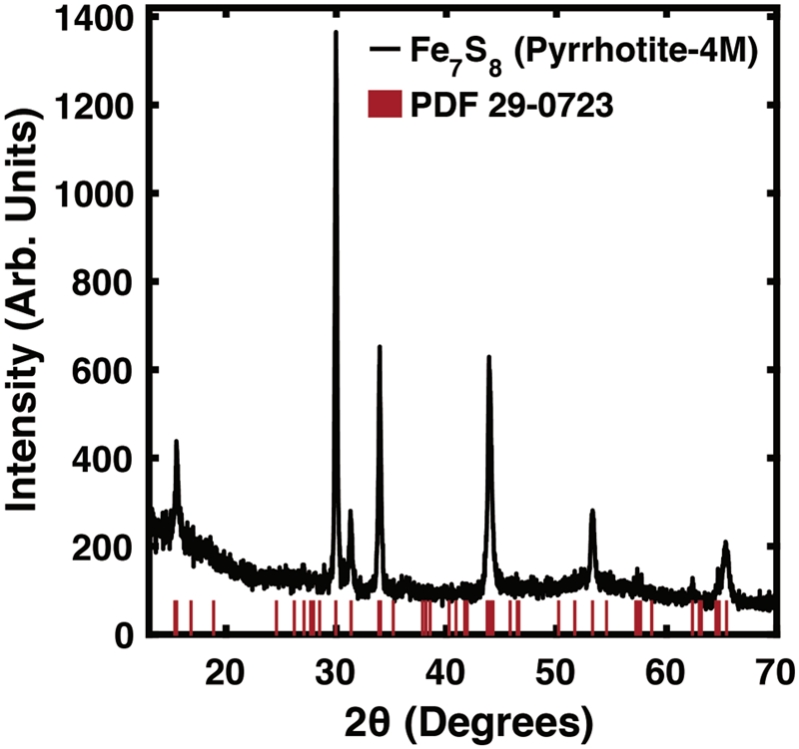
Diffraction pattern of the iron-sulfur reaction synthesized for seven hours. Red lines correspond to the location of diffraction peaks for the pyrrhotite reference pattern PDF 29-0723.

Structural characterization of the resulting FeS nanocrystals from a 400 min. synthesis at 180 °C is given in Fig. 4. A search of the ICDD database shows our synthetic material most closely indexes to PDF 29-0723, monoclinic pyrrhotite with a 4M superstructure, having lattice parameters *a* = 12.811 Å, *b* = 6.87 Å, and *c* = 11.885 Å. Rietveld refinement of laboratory XRD data for the purpose of more accurate lattice parameter determination yielded similar, but slightly expanded lattice constants of *a* = 12.836 Å, *b* = 6.882 Å, and *c* = 11.919 Å. Pyrrhotite systems of this structure are sometimes more broadly categorized as pyrrhotite “4 C” structures, although that designation does not distinguish the monoclinic phase.

While not all literature differentiates 4M and 4H pyrrhotite, there is reported crystallographic information on the overall 4C structure for both natural and synthetic samples^15,29,52–54^. Difficulty in distinction between 4M and 4H distortions has also been attributed to stacking faults in the material^11^. Monoclinic pyrrhotite-4M structures are generally denoted as having a stoichiometry of Fe_7_S_8_, or a 12.5% iron deficiency. However, iron deficiency for this superstructure is reportedly as low as 3.1% at 115 °C^15^. It should also be noted that an experimental diffraction pattern for pyrrhotite-4M is very close in construction to that of troilite, or stoichiometric FeS. Pyrrhotite-4M differs in that there is a higher density of diffraction peaks around the highest intensity peaks and that other minor distinguishing peaks are present. Clearly, careful treatment of XRD data should be considered when assessing this family of compounds.

### Growth and phase evolution in Fe-S compounds

**Figure 5:**
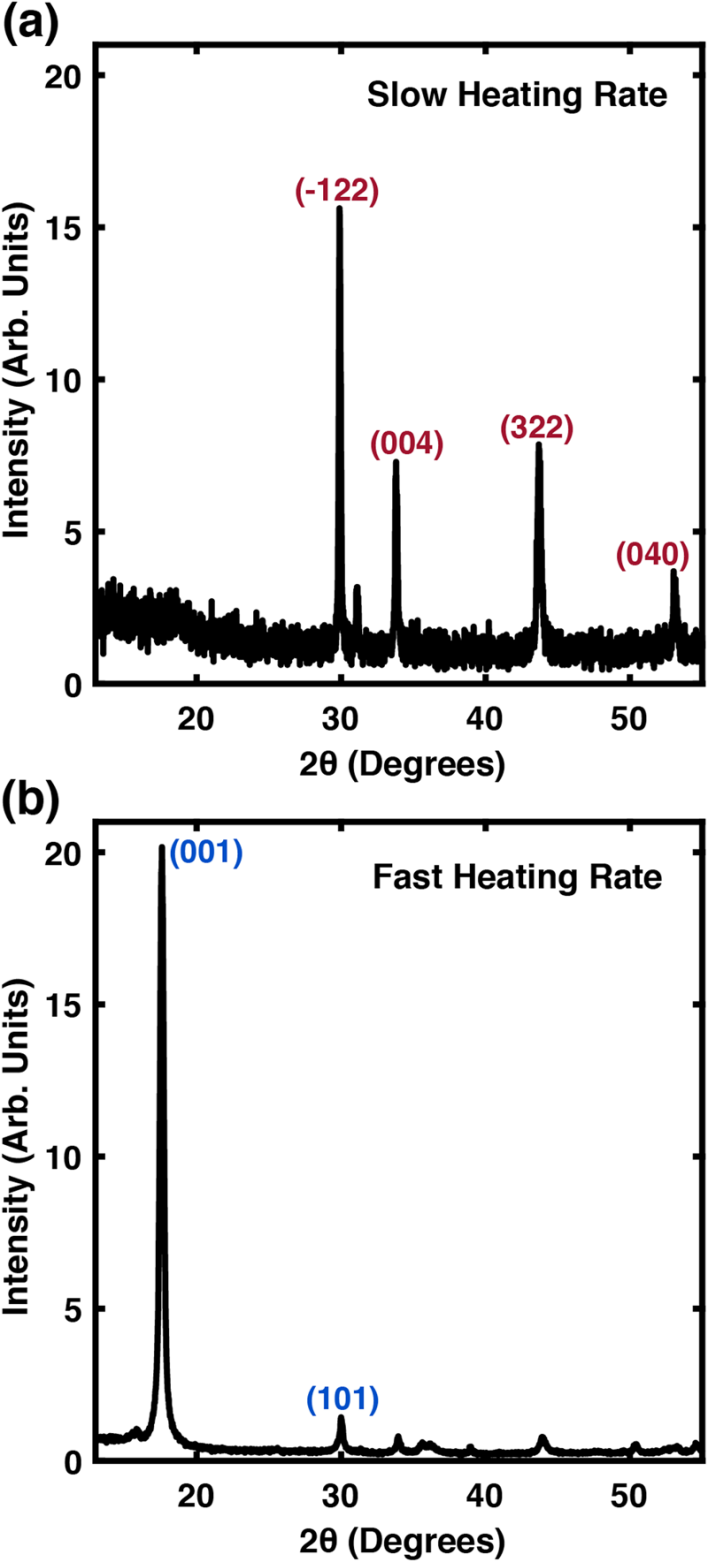
Diffraction patterns for the iron-sulfur reaction synthesized at different heating rates. Panel (a) contains a diffraction pattern corresponding to the slower rate, and shows diffraction peaks primarily associated with pyrrhotite (red). Panel (b) contains a diffraction pattern for a reaction product synthesized at a rate approximately 2× the first and shows diffraction peaks corresponding to mackinawite (blue).

The Fe-S synthesis outlined here is extremely sensitive to growth conditions, particularly heating rate. The heating rate, defined by the temperature ramp rate used to reach the final growth temperature from room temperature, was estimated to be 0.2 °C/min in the experiments described in Figs 2–4. Interestingly, by doubling the heating rate as shown in Fig. 5, we produced a product that was primarily mackinawite; heating at a roughly half this rate produces pyrrhotite-4M. These results occurred reproducibly in our system for experiments on both initial and equilibrium-length time scales. It has been suggested that the transition from tetragonal mackinawite to hexagonal pyrrhotite is related to changes in the symmetry of Fe-Fe bonds, which in two-dimensional mackinawite are short, strong bonds that induce puckering in sulfur layers^28^. Thus we posit that a faster heating rate affects synthetic pathway evolution and kinetically traps the mackinawite phase seen to emerge in this synthesis at nominally lower temperatures, thus inhibiting further transformation with increasing temperature^55^.

The onset of nucleation and growth, the temperatures at which phases form and transform, and the morphologies of the respective products in Fe-S system are sensitive to the chemical and thermodynamic environment in which they are formed. For Fe_1−x_S structures, monoclinic pyrrhotites are more stable below 200 °C than their hexagonal counterparts^5^. Exploration of the evolution of FeS synthesis under different reaction temperatures and times in a hydrothermal environment has shown smythite as a precursor to pyrrhotite formation. This represents a slightly more iron rich variant of the greigite intermediary observed in our experiments, and consequently the pyrrhotite end product in their work is more iron rich^29^ .The evolution time for a purely pyrrhotite product in this hydrothermal experiment is on a similar time scale relative to our anhydrous, ligand-based approach.

For a hydrothermal reaction system in which neither mackinawite nor sulfur presence is limited, Fe-S evolution at temperatures between 100 and 200 °C transform from mackinawite to FeS_2_ through greigite^51^. Hunger *et al*.^51^ demonstrated that formation and decay of these intermediaries follow zero-order kinetics; reaction progress at 175 °C and above (comparable to our growth temperatures) show the most rapid decay of mackinawite. Our system initially mimics this evolution pathway, however limitations from either precursor concentration or solvothermal kinetics provide us more iron-rich end products, with a Fe to S ratio of 7:8 as compared to the 1:2 ratio for iron pyrite.

Most other work on pyrrhotite kinetics investigates formation pathways at relatively low or high temperatures, which we summarize here to put our work into context with syntheses of the surrounding temperature regimes. High temperature work by Lennie *et al*.^56^ demonstrates the transformation to pyrrhotite from mackinawite *via* solid-state diffusion at 530–545 K. They note that the hexagonal pyrrhotite phase is “kinetically limited” in the sense that its rate of formation from mackinawite by solid-state diffusion is extremely slow. Evolution of stoichiometric FeS at synthesis temperatures of less than 100 °C are reported as having no intermediaries while progressing to FeS_2_; these pathways are understood to be the result of non-zero order reactions with H_2_S or polysulfides and to be kinetically limited by the concentration of a solid educt^57^.

A solvothermal synthesis of greigite by Yuan *et al*.^58^ showed the importance of excess sulfur in the formation of greigite, wherein reactant ratios at and near 1:1 Fe to S yield Fe_7_S_8_ in the 3T superstructure. While they do not discuss the intermediaries of transformation, our results are consistent in that stoichiometric addition of Fe:S precursors results in an iron deficient FeS structure. Additionally, their work demonstrates that choice of iron precursor for a given reaction condition plays a major role in synthetic outcome; thus, it seems likely that the 2+ oxidation state of Fe resulting from our precursor choice plays a central role in the formation of our end compound.

While hydrothermal reactions can utilize the role of water in emulating more natural processes, solvothermal reactions allow a more careful manipulation of growth kinetics. Precursors, solvents, other ligands, and temperature can be chosen to control system evolution by considering constituent interactions with regard to the reactivity, stability, and solvation^59,60^. As such we present a synthetic environment with 1:1 ratio of precursors reacted in OLA, yielding a reactive environment in which iron is in the 2+ state and a plate-like morphology is produced. With this as a starting point, future work will focus on ligand selection in the Fe-S system for a given iron content or morphology, comparable to what is seen in the pyrite system^48,61^.

### Computational insights on near stoichiometric troilite and pyrrhotite

Given how important bulk vacancies are in determining the properties of iron sulfides^62^, it is surprising that they have not received more attention in the computational literature^21–23^. In this paper, we start with troilite, the stoichiometric end group of the pyrrhotite family. This is a reasonable approach because both troilite and the pyrrhotites are based on the NiAs crystal structure, and there may be a near continuum of stable structures between defective troilite and stable pyrrhotites.

**Figure 6:**
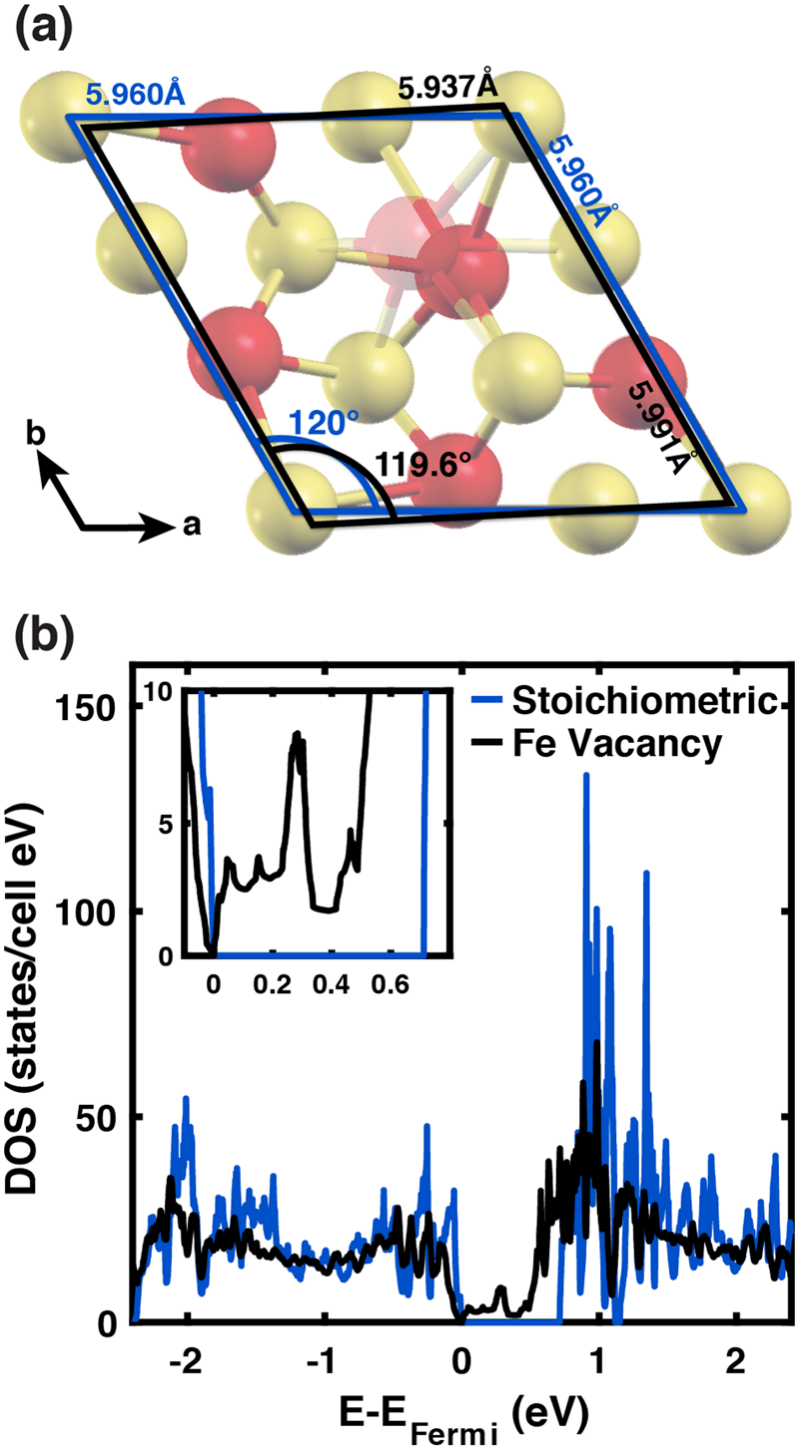
Structure and electronic deviations in FeS with and without a Fe vacancy as determined by DFT. Panel (a) shows a shift from hexagonal (blue) to monoclinic (black) geometry with removal of an iron atom, where iron and sulfur are labeled red and yellow, respectively. The density of states for FeS with and without an iron vacancy is illustrated in (**b**) by black and blue curves, respectively and shows a decrease in band gap from 0.71 eV to 0 eV with the introduction of a Fe vacancy.

Figure 6b shows the DOS for the stoichiometric and Fe-vacant structures. With no defects, the troilite unit cell maintains its *P-62c* symmetry and is insulating with a band gap of 0.71 eV. This band gap is larger than that reported in recent DMFT calculations^40,41^, but similar to that reported in another DFT + U paper^8^. Once we introduce a single neutral iron vacancy, the structure distorts from hexagonal into a monoclinic cell resembling a pyrrhotite phase with *a* = 5.937 Å, *b* = 5.991 Å, *c* = 11.655 Å, and unit cell volume = 359.52 Å^3^, shown in Fig. 6a. We define this fully relaxed, monoclinic structure as iron deficient troilite.

This structural change is insensitive to which iron atom gets removed, suggesting that our computational realizations of iron deficient troilite is similar to what one might call a type of pyrrhotite with the atomic formula Fe_11_S_12_. Although iron deficient troilite resembles a pyrrhotite structure more closely than troilite, we cannot classify it as such since it does not have the same stoichiometry as pyrrhotite-4M (Fe_7_S_8_) and does not share crystal symmetries with the Fe_11_S_12_ polytype pyrrhotite-6C. Since iron deficient troilite is a fully relaxed structure, it supports the presence of stable intermediates existing between troilite and pyrrhotite. Our results suggest that iron defects cause a local structural distortion in troilite, but these distortions have a correlation length that is short on the scale of an individual unit cell. If the results for the crystal structure or density of states depended strongly on which Fe atom was removed, then our computational model for an iron-deficient structure would represent a periodically defective structure that would not be a faithful model for experimental crystal structures whose vacancies appear more randomly.

The band gap of the defective structure completely closes and remains ungapped as U goes from 0 to 1.4 eV, which shows robustness with respect to the parameterization of U. One may think of the gap closing due to the appearance of midgap states clustering into an already narrow band. In solids with wider gaps, disorder-induced midgap states lead to so-called “Urbach tails” in the absorption spectrum^63,64^.

To further investigate the relationship between troilite and pyrrhotite, we calculated the DOS of pyrrhotite-4M. As shown in Fig. 2b, the pyrrhotite-4M unit cell is part of the monoclinic space group *C2/c* with four formula units of Fe_7_S_8_ in the unit cell. Using the experimental structure from Powell *et al*.^11^ with U* = 1.4 eV and fixing the atomic positions, the DOS is gapless, just like iron deficient troilite.

## Conclusions

In this work, we studied synthetic pyrrhotite-4M nanocrystals and investigated the relationship of this compound with its stoichiometric end member, troilite, through electronic structure calculations. While some previous studies suggested that the morphology and composition of Fe-S solids is complex and sensitive to preparation protocol, our work clearly demonstrates that the product state distribution of crystal structures is under kinetic, not thermodynamic, control. We utilized an amine-driven synthetic route, seldom used for pyrrhotite growth, to develop an understanding of a solvothermally derived Fe-S process and the resulting solids, with a focus on the moderately defective compositions approaching Fe_7_S_8_. Computationally, we showed the role that Fe-defects have on troilite and predict that any Fe-vacancy introduced into the stoichiometric end member changes the unit cell, transforming the hexagonal crystal structure of troilite into a monoclinic structure that is a signature in a number of pyrrhotite structures. Our calculations also predict that small concentrations of iron defects erase the band gap in troilite by introducing midgap defect states in an already narrow gap. It is possible, or even likely, that the crystal structures reported for experimental troilite samples in fact more closely resemble these defective structures. A similar phenomenon has been reported in iron pyrite, where atomic defects, either in the bulk or on the surface, narrow the gap substantially^21–23^. Given that iron-sulfur based compounds are poised to have a tremendous impact in technologies including energy storage^65^, photovoltaics^48^, high temperature superconductors^7,9,24^, and catalysis^66^, it becomes evident that more research is required to determine structure-property relationships in these materials, particularly with regard to even small degrees of off-stoichiometry.
